# Involvement of Enteric Glia in Small Intestine Neuromuscular Dysfunction of Toll-Like Receptor 4-Deficient Mice

**DOI:** 10.3390/cells9040838

**Published:** 2020-03-31

**Authors:** Silvia Cerantola, Valentina Caputi, Ilaria Marsilio, Manuela Ridolfi, Sofia Faggin, Michela Bistoletti, Cristina Giaroni, Maria Cecilia Giron

**Affiliations:** 1 Department of Pharmaceutical and Pharmacological Sciences, University of Padova, 35131 Padova, Italy; silvia.cerantola.2@gmail.com (S.C.); valekap@gmail.com (V.C.); ilaria.marsilio@gmail.com (I.M.); manuelaridolfi95@gmail.com (M.R.); sofia.faggin95@gmail.com (S.F.); 2 San Camillo Hospital, 31100 Treviso, Italy; 3 APC Microbiome Ireland, University College Cork, IE5 Cork, Ireland; 4 Department of Medicine and Surgery, University of Insubria, 21100 Varese, Italy; michelabistoletti@gmail.com (M.B.); cristina.giaroni@uninsubria.it (C.G.)

**Keywords:** enteric nervous system, enteric glial cells, fluoroacetate, innate immunity, intestinal motility, knockout mice, small intestine, toll-like receptor 4

## Abstract

Enteric glial cells (EGCs) influence nitric oxide (NO)^−^ and adenosine diphosphate (ADP)^−^ mediated signaling in the enteric nervous system (ENS). Since Toll-like receptor 4 (TLR4) participates to EGC homoeostasis, this study aimed to evaluate the possible involvement of EGCs in the alterations of the inhibitory neurotransmission in TLR4^−/−^ mice. Ileal segments from male TLR4^−/−^ and wild-type (WT) C57BL/6J mice were incubated with the gliotoxin fluoroacetate (FA). Alterations in ENS morphology and neurochemical coding were investigated by immunohistochemistry whereas neuromuscular responses were determined by recording non-adrenergic non-cholinergic (NANC) relaxations in isometrically suspended isolated ileal preparations. TLR4^−/−^ ileal segments showed increased iNOS immunoreactivity associated with enhanced NANC relaxation, mediated by iNOS-derived NO and sensitive to P2Y1 inhibition. Treatment with FA diminished iNOS immunoreactivity and partially abolished NO^−^ and ADP^−^ mediated relaxation in the TLR4^−/−^ mouse ileum, with no changes of P2Y1 and connexin-43 immunofluorescence distribution in the ENS. After FA treatment, S100β and GFAP immunoreactivity in TLR4^−/−^ myenteric plexus was reduced to levels comparable to those observed in WT. Our findings show the involvement of EGCs in the alterations of ENS architecture and in the increased purinergic and nitrergic-mediated relaxation, determining gut dysmotility in TLR4^−/−^ mice.

## 1. Introduction

The enteric nervous system (ENS), like the central nervous system (CNS), is composed of neurons and enteric glial cells (EGCs). EGCs share several morphological, molecular and functional similarities with CNS astrocytes. Enteric glia surrounds neurons exerting several neurotrophic, neuroprotective, and neuro-immunomodulatory functions to protect neuronal networks and ensuring gut functions [1,2,3,4,5]. Therefore, EGCs are considered the central regulators of ENS homeostasis and the disruption of glial functions has been implicated in several gastrointestinal disorders [1], such as functional gastrointestinal disorders, inflammatory bowel diseases (IBDs) [6,7], chronic idiopathic intestinal pseudo-obstruction, diverticular disease [8], necrotizing enterocolitis [9], Chagas disease [10], type II diabetes [11], and Parkinson’s disease [12].

The expression of a common set of biomarkers, including S100β [13], glial fibrillary acidic protein (GFAP) [14], and the transcription factor, SRY-related HMG-box (Sox) 10 [2], reflects the origin of EGCs from neural crest-derived progenitors [15]. Since the mature EGC phenotype is determined by the distinctive milieu in which it resides, extensive glial diversity occurs within the various layers of the gut wall and can be revealed by specific marker signatures [16]. The neural crest marker Sox10 is the most widely expressed in ECGs, although none of the three markers exhibit a pan-expression in EGCs [17]. The EGCs, like all glial cells, are highly active and may undergo plastic changes in response to harmful stimuli, for example during an inflammatory injury, characterized by “reactive gliosis” associated to overexpression of GFAP or S100β [2,18,19].

Derangement of the ENS architecture during gut neuropathies may underlie development of functional bowel disorders, often exhibiting an unbalanced equilibrium between excitatory and inhibitory neurotransmission. A dysregulated neurotransmission affects enteric reflexes which are finely tuned by the cross signaling between enteric neurons and glia. The expression of receptors for multiple neurotransmitters, not only in neurons but also in glial cells, ensures the correct modulation of neural circuits, and the consequent regulation of motility and secretion. Furthermore, EGCs activation by harmful signals is often the driver of neurodegenerative processes during intestinal inflammation [20]. Therefore, the elucidation of the events promoting EGCs activation and the possible consequences on neurotransmission pathways is important to understand gut functional regulation [21].

Fluoroacetate (FA) and its metabolite fluorocitrate (FC) [22] are toxins preferentially taken up by glial cells both in the CNS and ENS, where both molecules specifically inhibit the enzymatic activity of aconitase, determining the block of Krebs cycle [22,23,24]. Therefore, these gliotoxins have been widely used as experimental tools to probe glial activity and to assess the involvement of EGCs in functional bowel disorders [23,24,25,26].

In the gut, Toll-like receptor 4 (TLR4) is expressed in both EGCs and neurons and mediates enteric neuronal survival [27,28] and host-immune responses. Recently, we have shown that the absence of TLR4 causes alterations in ileal neuromuscular function and glial phenotype leading to ileal dysmotility and reactive gliosis [29] However, a clear-cut correlation between neuromuscular dysfunction and alterations of EGCs function in TLR4^−/−^ mice has not yet been demonstrated. Thus, to better understand the role of enteric glia in the gastrointestinal anomalies observed in TLR4^−/−^ mice, we treated isolated ileal segments with the gliotoxin FA in order to determine whether the disruption of EGCs by FA could restore the function and the structure of ileal ENS in TLR4^−/−^ mice.

## 2. Materials and Methods

### 2.1. Animals

Experiments were performed using male TLR4^−/−^ (B6.B10ScN-Tlr4^lps-del^/JthJ; 9 ± 1 weeks old) and sex- and age-matched wild type (WT) C57BL6/J mice (Jackson Laboratories, Bar Harbor, ME, USA). Animals were pathogen-free and were housed in ventilated cages (IVC) at the animal facility of the Department of Pharmaceutical and Pharmacological Sciences, University of Padova, Italy. All animals were kept under controlled environmental conditions (temperature 22 °C and 50% humidity) with a light/dark cycle of 12 h, free access to food (standard chow diet) and tap water. All procedures were approved by the Italian Ministry of Health (authorization number: 1142/2015-PR), the Animal Care and Use Ethics Committee and University of Padova and were conducted in accordance with ARRIVE guidelines [30,31] and with national and EU guidelines for the handling and use of experimental animals. A total of 72 mice (i.e., 36 mice for each transgenic group) were studied in the following experiments.

### 2.2. Fluoroacetate Treatment

To assess the impact of enteric glia on ENS architecture and function, distal ileum segments from WT and TLR4^−/−^ mice were incubated for 1 h with 10 µM FA at 37 °C with 95% O_2_/5% CO_2_. Ileal samples were subjected to neuromuscular function studies and their adjacent tissue segments were used for IHC and quantitative RT-PCR experiments [26].

### 2.3. Neuromuscular Function Studies

Relaxation studies were performed as previously described [32,33]. Animals were sacrificed by cervical dislocation. All the following analytical procedures were conducted blindly. The abdomen was immediately opened, the ileum was excised and quickly transferred into a Petri dish filled with warm (37 °C) Krebs solution (NaCl 118 mM, KCl 4.7 mM, CaCl_2_∙2H_2_O 2.5 mM, MgSO_4_∙7H_2_O 1.2 mM, K_2_HPO_4_ 1.2 mM, NaHCO_3_ 25 mM, C_6_H_12_O_6_ 11 mM). After gently flushing ileal contents, full-thickness 1 cm-distal ileum segments were isolated, mounted along the longitudinal axis in 10-mL-organ baths and allowed to equilibrate for 30 min in Krebs solution maintained at 37 °C with 95% O_2_/5% CO_2_. Mechanical activity of ileum segments was recorded by isometric transducers (World Precision Instruments, Berlin, Germany) connected to a PowerLab 4/30 system (ADInstruments, Oxford, UK). After 1 h-incubation with or without 10 µM FA, dissolved in Krebs solution, neuronal-mediated relaxations were analyzed following electrical field stimulation (EFS; 10 Hz; 40 V) in non-adrenergic non-cholinergic (NANC) conditions, obtained by adding 1 μM guanethidine and 1 μM atropine to the organ baths. To evaluate nitrergic-mediated inhibitory neurotransmission, 10 Hz-EFS was performed under NANC conditions in ileal segments pre-incubated for 20 min with 100 μM Nω-nitro-l-arginine methyl ester (L-NAME), a pan-nitric oxide synthase (NOS) inhibitor, or 10 μM 1400 W, a selective inducible NOS (iNOS) inhibitor. In a second series of experiment, the purinergic component of the relaxant response was investigated by performing 10 Hz-EFS under NANC conditions in ileal segment pre-incubated for 20 min with 1 μM MRS2500, a P2Y1 receptor antagonist. In NANC conditions, EFS determined a primary on-relaxation of the smooth muscle of ileum segments, which was calculated as area under the curve (AUC) and normalized to gram of dry tissue weight to allow comparisons between tissues samples [33].

### 2.4. Immunohistochemistry on Ileal whole Mount Preparations

Immunohistochemistry studies were performed as previously described [29,33]. To assess changes in neuro-glial plasticity on ENS architecture, fresh isolated distal ileum 10 cm-segments were gently flushed with warm (37 °C) Krebs solution, to remove any luminal content and subjected to 1 h-incubation with or without 10 µM FA, dissolved in Krebs solution. Ileal segments were then rinsed with PBS and exposed to fixative solution (4% PFA in PBS) for 2 h at room temperature. After 3 × 15 min washes in PBS, ileal segments were cut in 0.5 cm-pieces opened along the mesenteric border and placed as a flat sheet to the bottom of Sylgard-coated dishes with the mucosal side down. Using a dissecting microscope, tissues were separated into two layers: the outer musculature with adhering serosa and the submucosa/mucosa. The circular muscle was removed to yield whole mounts of longitudinal muscle with attached myenteric plexus (LMMPs) [34,35,36]. LMMPs preparations were gently stretched and pinned down on the bottom of Sylgard-coated dishes and washed in PBT (PBS with 0.3% Triton X-100) for 45 min with gentle shaking. After blocking nonspecific sites with PBT containing 2% BSA for 1.5 h at room temperature, LMMPs were incubated overnight at room temperature with the primary antibodies (Table 1) diluted in PBT and BSA 2%. The following day, LMMPs preparations were washed and incubated for 2 h at room temperature with respective secondary antibodies (Table 1) diluted in PBT and BSA 2%. LMMPs preparations were mounted on glass slides using a Mowiol Mounting Medium (100-mM Tris-HCl (pH 8.5), 9% Mowiol 4–88, 25% glycerol and 0.1% DABCO) and stored at –20 °C in the dark until analysis. Negative controls were obtained by incubating sections with isotype-matched control antibodies at the same concentration as primary antibody and/or pre-incubating each antibody with the corresponding control peptide (final concentration as indicated by manufacturer’s instructions).

### 2.5. Confocal Image Acquisition and Analysis

Images were acquired using a Zeiss LSM 800 confocal imaging system (Oberkoken, Germany) equipped with an oil-immersion 63× objectives (NA 1.4). Z-series images (25 planes for LMMP whole mount preparations) of 1024 × 1024 pixels were processed as maximum intensity projections. All microscope settings were kept constant for all images. Fluorescence intensity (density index) of GFAP, S100β, SOX10, connexin43 and of P2Y1 receptor, iNOS was assessed for each antigen by capturing 20 images per mouse, as reported previously [26,33]. The intensity of staining for each antibody was expressed as the density index of labelling per myenteric ganglion area [11] and was reported as mean ± SEM. The analysis of the total neuron population was performed by counting HuC/D^+^ cells in 10 randomly chosen images per mouse. The total number of HuC/D^+^ neurons was recorded in each image and normalized per myenteric ganglion area, as previously described [11,33,37]. To evaluate the distribution of nitrergic neurons in ileal myenteric plexus, the number of nNOS^+^ enteric neurons was blindly counted in 10 randomly chosen images per mouse and normalized per myenteric ganglion area.

### 2.6. RNA Isolation and Quantitative RT-PCR

Total RNA was extracted from small intestine wall segments after removing the mucosa, as described by Bistoletti et al., (2019) [36]. cDNA was obtained by retrotranscribing 2 µg of total RNA using the High Capacity cDNA synthesis kit (Applied Biosystems, Milan, Italy). Quantitative RT-PCR was performed on the Abi Prism 7000 real-time thermocycler (Applied Biosystems, Milan, Italy) with Power Sybr Green Universal PCR Master Mix (Applied Biosystems, Milan, Italy) according to the manufacturer’s instructions. Primers were designed using Primer Express software (Applied Biosystems, Milan, Italy) as reported in Table 2 and a final concentration of 500 nM for each primer was used. Primers were designed to have a similar amplicon size and similar amplification efficiency as required for applying the 2^−ΔΔCt^ method to compare gene expression in the intestinal preparations of WT and TLR4^−/−^ mice in the presence or absence of FA, with respect to values obtained in WT [36]. β-actin was used as housekeeping gene.

### 2.7. Chemicals

Unless otherwise specified, all chemicals were obtained from Sigma–Aldrich (Milan, Italy) and were of the highest commercially available analytical grade. PFA was purchased from Electron Microscopy Sciences-Società Italiana Chimici (Rome, Italy), and Triton-X-100 was obtained from Applichem (Milan, Italy).

### 2.8. Statistical Analysis

Data were analyzed using GraphPad Prism 3.03 (San Diego, CA, United States) and are expressed as mean ± SEM. The distribution of data was tested with the Shapiro-Wilk normality test. Differences between the experimental groups were assessed using paired or unpaired Student’s t-test and one-way or two-way analysis of variance (ANOVA), followed by post-hoc Bonferroni test. The results were considered statistically significant at *p* < 0.05; “*N*” indicates the number of animals.

## 3. Results

### 3.1. In Vitro Fluoroacetate Treatment Reduces Enteric Reactive Gliosis in TLR4^−/−^ Mice

**Figure 1:**
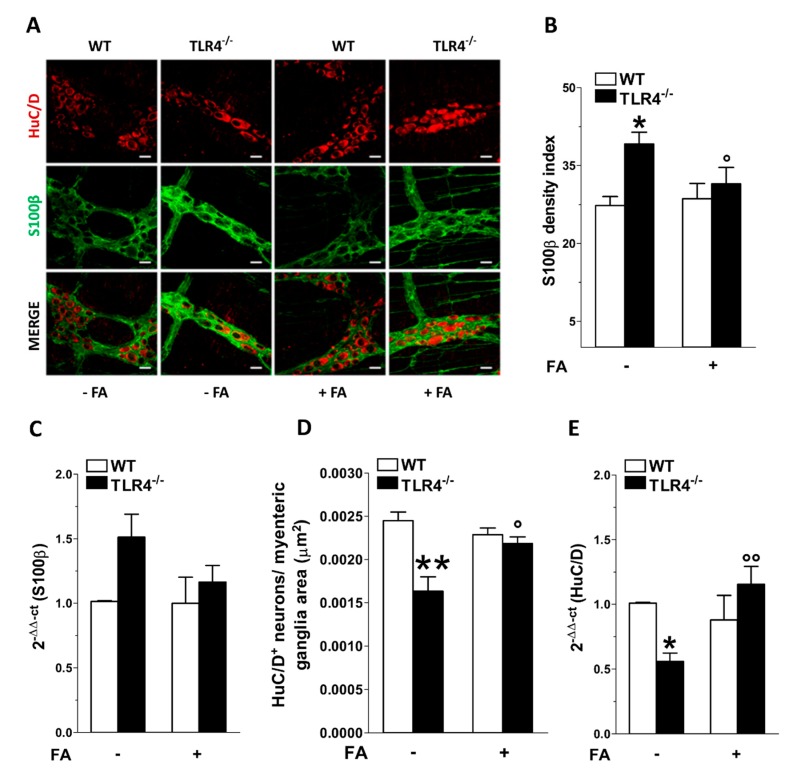
Effect of fluoroacetate (FA) treatment on myenteric ganglia neural and glial cells. (**A**) Representative confocal microphotographs showing the distribution of S100β (green) and HuC/D (red) in small intestine LMMPs of WT and TLR4^−/−^ mice in the presence or absence of 10 μM FA (bars = 22 μm). (**B**,**C**) Analysis of S100β density index in ileal myenteric ganglia (**B**) and S100β mRNA levels in small intestine segments (**C**) of WT and TLR4^−/−^ mice in the presence or absence of 10 μM FA. (**D**,**E**) Total number of HuC/D^+^ neurons in ileal myenteric ganglia (**D**) and HuC/D mRNA levels in small intestine segments (**E**) of WT and TLR4^−/−^ mice in the presence or absence of 10 μM FA. Data are reported as mean ± SEM for all panels. * *P* < 0.05, ** *P* < 0.01 vs. WT; ° *P* < 0.05, °° *P* < 0.01 vs. respective control without FA; *N* = 6 mice/group.

**Figure 2:**
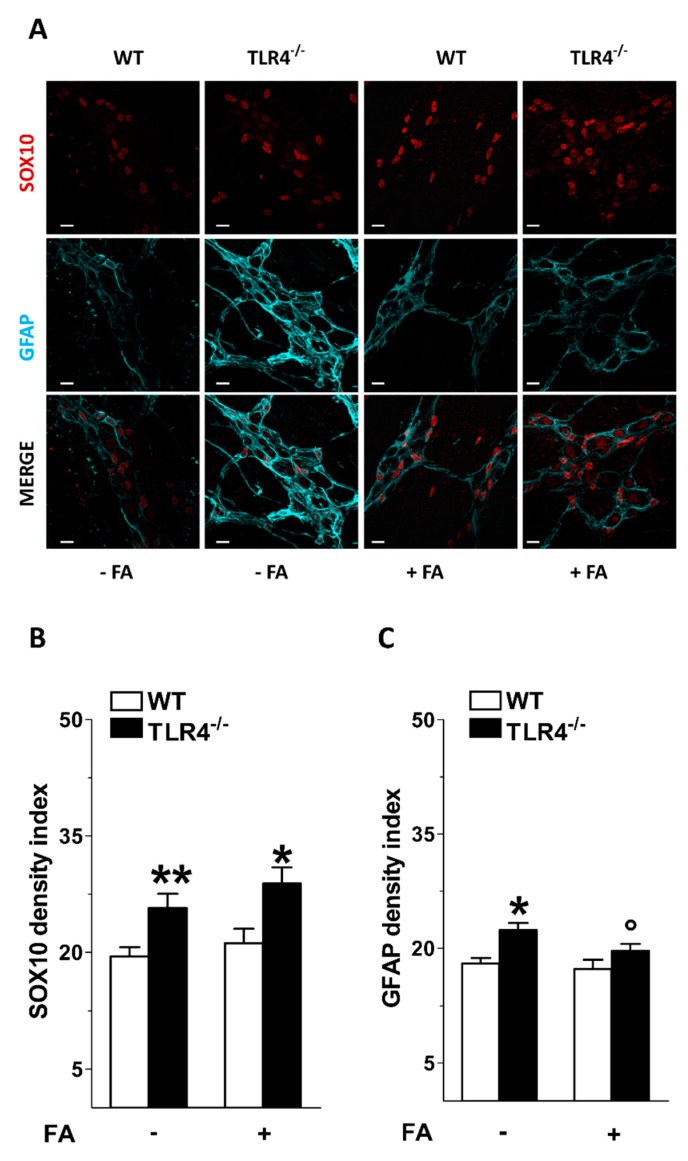
Effect of fluoroacetate (FA) treatment on enteric glial phenotype. (**A**) Representative confocal microphotographs showing the distribution of SOX10 (red) and GFAP (cyan) in ileal LMMPs of WT and TLR4^−/−^ mice in the presence or absence of 10 μM FA (bars = 22 μm). (**B**,**C**) Analysis of SOX10 and GFAP density index in ileal LMMPs of WT and TLR4^−/−^ mice in the presence or absence of 10 μM FA. Data are reported as mean ± SEM for all panels. * *P* < 0.05, ** *P* < 0.01 vs. WT; ° *P* < 0.05 vs. respective control without FA; *N* = 6 mice/group.

In the myenteric plexus of TLR4^−/−^ mouse ileum, the immunoreactivities of glial markers S100β, SOX10 and GFAP were significantly higher compared to those found in WT mice (+49 ± 1%; +25 ± 2%; +15 ± 4%, respectively; Figure 1 and Figure 2). The total number of HuC/D^+^ neurons was significantly lower (−20 ± 4%; Figure 1A,D), and was reflected by lower levels of HuC/D transcripts ileal segments of TLR4^−/−^ mice compared to WT mice (−45 ± 1% Figure 1A,D,E). Treatment with FA restored S100β immunofluorescence density and total number of HuC/D^+^ myenteric neurons to levels comparable to WT mice as confirmed by S100β and HuC/D mRNA levels (Figure 1B–E).

In the LMMPs of TLR4^−/−^ mice, treatment with FA resulted in a significant reduction in GFAP immunoreactivity with no changes in SOX10 density index (Figure 2).

### 3.2. Influence of Enteric Glial Cells (EGCs) on Nitrergic Neurotransmission in Small Intestine of TLR4^−/−^ Mice

**Figure 3:**
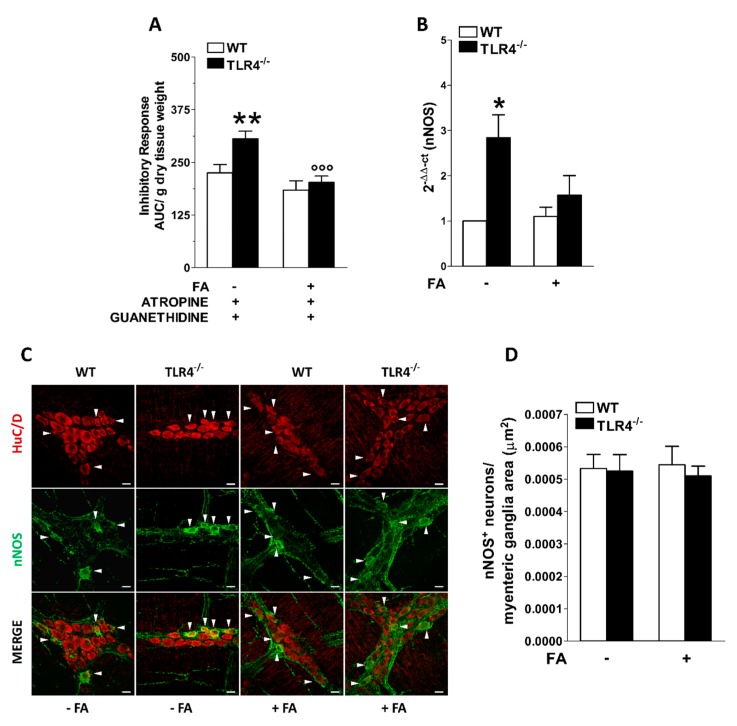
Involvement of EGCs in the modulation of nitrergic neurotransmission of small intestine in TLR4^−/−^ mice. (**A**) 10 Hz EFS-evoked NANC relaxation responses in ileal segments of WT and TLR4^−/−^ mice in the presence or absence of 10 μM FA. (**B**) qRT-PCR quantification of nNOS mRNA levels in small intestine segments of WT and TLR4^−/−^ mice in the presence or absence of 10 μM FA. (**C**) Representative confocal microphotographs showing the distribution of nNOS^+^ (green) and HuC/D^+^ (red) neurons in ileal LMMPs of WT and TLR4^−/−^ mice in the presence or absence of 10 μM FA (bars = 22 μm). White arrowhead point to HuC/D^+^ nNOS^+^ neurons. (**D**) Number of nNOS^+^ neurons in ileal LMMPs of WT and TLR4^−/−^ mice in the presence or absence of 10 μM FA. Data are reported as mean ± SEM for all panels. * *P* < 0.05, ** *P* < 0.01 vs. WT; °°° *P* < 0.005 vs. respective control without FA; *N* = 6 mice/group.

In NANC conditions, EFS at 10 Hz caused a 1.35-fold increase in the relaxation of TLR4^−/−^ ileal segments together with a 2.8-fold increase in nNOS mRNA levels with no changes in nNOS^+^ myenteric neurons (Figure 3). In TLR4^−/−^ mice, the disruption of EGCs activity by in vitro incubation with FA restored an inhibitory response and nNOS mRNA transcripts were comparable to those obtained in WT mice (Figure 3A,B).

**Figure 4:**
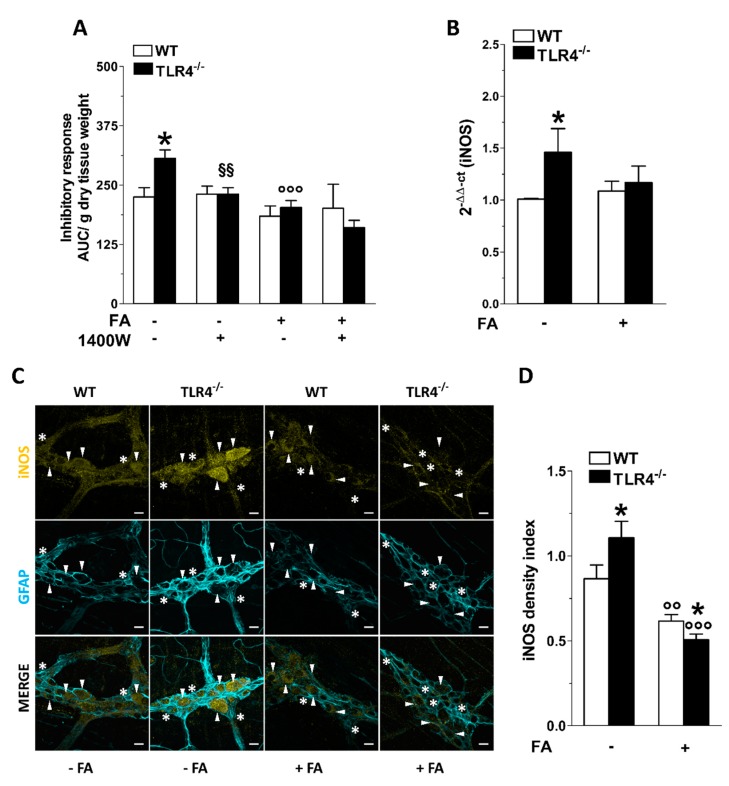
TLR4 signaling and EGC activity influence NO-mediated relaxation. (**A**) 10 Hz EFS-evoked relaxation in NANC conditions with or without 10 μM FA or 10 μM 1400 W (iNOS inhibitor) in ileal segments of WT and TLR4^−/−^ mice. (**B**) qRT-PCR quantification of iNOS mRNA levels in small intestine segments of WT and TLR4^−/−^ mice in the presence or absence of 10 μM FA. (**C**) Representative confocal microphotographs showing the distribution of iNOS (yellow) and GFAP (cyan) and (**D**) analysis of iNOS density index in ileal LMMPs of WT and TLR4^−/−^ mice in the presence or absence of 10 μM FA (bars = 22 μm). White arrowheads indicate iNOS^+^ neurons, stars indicate iNOS^+^ glial cell bodies, respectively. Data are reported as mean ± SEM for all panels. * *P* < 0.05 vs. WT; °° *P* < 0.01, °°° *P* < 0.001 vs. respective control without FA; §§ *P* < 0.01 vs. respective control without 1400 W; *N* = 6 mice/group.

Considering the involvement of iNOS in sustaining part of the NO-mediated relaxation in the gut and given the enhanced immunoreactivity of iNOS in TLR4^−/−^ myenteric ganglia [29], we assessed whether the disruption of the EGCs activity could affect iNOS-mediated inhibitory response. Following iNOS inhibition with 1400 W, the relaxant response of ileal segment from TLR4^−/−^ mice resulted comparable to that of WT mice (Figure 4A). Higher expression of iNOS in ileal specimens of TLR4^−/−^ mice was evidenced by qRT-PCR (+42 ± 3%; Figure 4B). Interestingly, the inhibitory response of ileal segments treated with FA in the presence or absence of 1400 W was comparable between genotypes (Figure 4A), suggesting that in absence of TLR4, NO release is mostly mediated by iNOS expressed by EGCs [24,29] (Figure 4C,D). Accordingly, the disruption of EGCs activity with FA determined a marked reduction of iNOS immunoreactivity and mRNA levels in both genotypes, as shown in Figure 4B–D.

### 3.3. Inhibition of Enteric Glial Cells (EGC) Metabolism Affects Purinergic-Mediated Inhibitory Response of TLR4^−/−^ Ileal Segments

**Figure 5:**
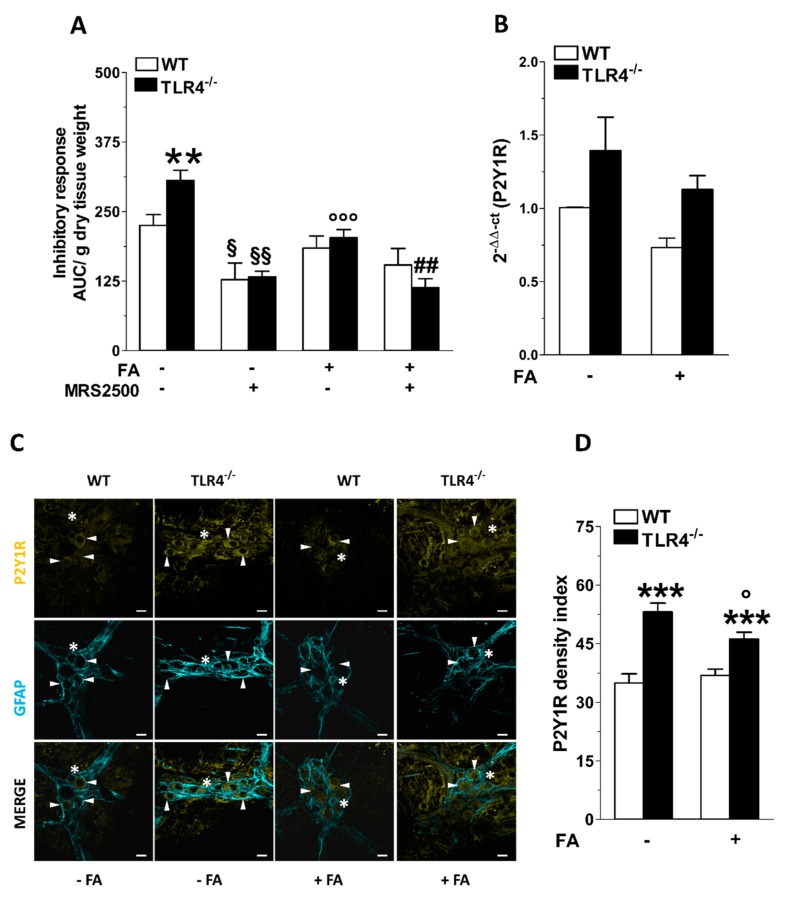
TLR4 signaling and EGC activity affect P2Y1 receptor-mediated small intestinal relaxation. (**A**) 10 Hz EFS-evoked relaxation in NANC conditions with or without 10 μM FA or 10 μM MRS2500 (P2Y1R inhibitor) in ileal segments of WT and TLR4^−/−^ mice. (**B**) qRT-PCR quantification of P2Y1R mRNA levels in small intestine segments of WT and TLR4^−/−^ mice in the presence or absence of 10 μM FA. (**C**) Representative confocal microphotographs showing the distribution of P2Y1R (yellow) and GFAP (cyan) and (**D**) analysis of P2Y1R density index in ileal LMMPs of WT and TLR4^-/-^ mice in the presence or absence of 10 μM FA (bars = 22 μm). White arrowheads indicate P2Y1^+^ neurons, stars indicate P2Y1^+^ glial cell bodies, respectively. Data are reported as mean ± SEM for all panels. ** *P* < 0.01, *** *P* < 0.001 vs. WT; ° *P* < 0.05, °°° *P* < 0.001 vs. respective control without FA; § *P* < 0.05, §§ *P* < 0.01 vs. respective control without MRS2500; ## *P* < 0.01 vs. respective control with FA and without MRS2500; *N* = 6 mice/group.

We have previously shown that TLR4^−/−^ mice display a marked increase of P2Y1 receptor (P2Y1R) immunoreactivity in myenteric ganglia and higher ADP-mediated relaxation sensitive to the P2Y1R selective antagonist MRS2500 [29,38]. Therefore, we sought to determine the impact of EGCs activity on purinergic-mediated relaxation in NANC conditions in absence or in presence of FA. The addition of MRS2500 in NANC conditions determined a reduction in the inhibitory response of TLR4^−/−^ ileal segments (-55 ± 2%), resulting in a response comparable to that of WT mice (Figure 5A). The combination of FA and MRS2500 determined a reduction of NANC-mediated relaxation similar to that obtained with MRS2500 alone, in both WT and TLR4^−/−^ mice (Figure 5A). qRT-PCR data showed no changes of P2Y1R mRNA transcripts in TLR4^−/−^ ileal tissue that was not affected by FA treatment (Figure 5B). The increased immunoreactivity of P2Y1R (+41 ± 3%), shown in the myenteric plexus of TLR4^−/−^ mice ileum, was partially blunted by FA treatment, and remained significantly higher compared to that of WT (Figure 5C,D).

**Figure 6:**
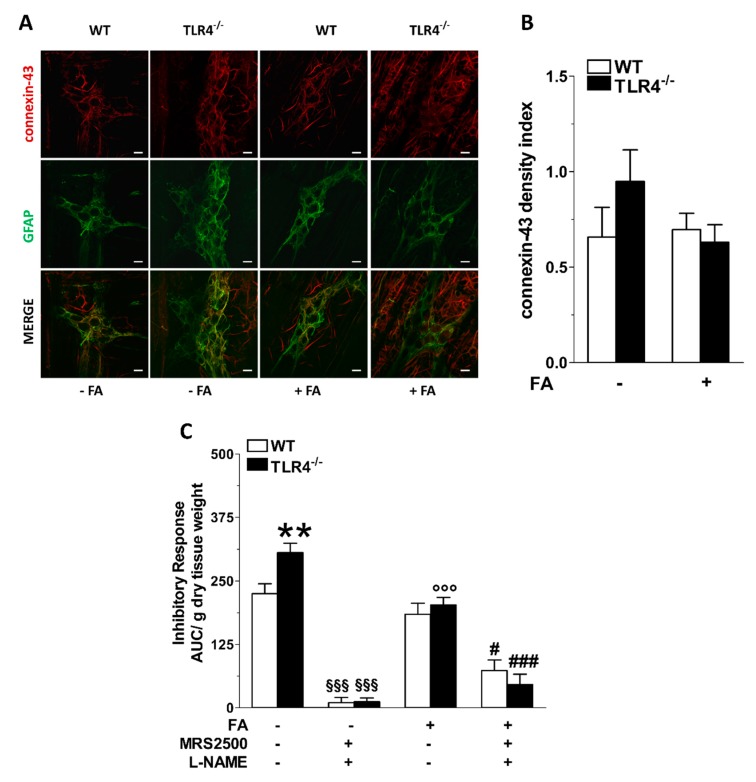
TLR4 signaling and EGC activity regulate nitrergic- and purinergic-mediated inhibitory response of the small intestine. (**A**) Representative confocal microphotographs showing the distribution of connexin-43 (red) and GFAP (green) and (**B**) analysis of connexin-43 density index in ileal LMMPs of WT and TLR4^−/−^ mice in the presence or absence of 10 μM FA (bars = 22 μm). (**C**) 10 Hz EFS-evoked relaxation in NANC conditions with or without 10 μM FA or 10 μM MRS2500 (P2Y1R inhibitor) or 1 μM L-NAME (pan-NOS inhibitor) in WT and TLR4^−/−^ mice. Data are reported as mean ± SEM for all panels. ** *P* < 0.01 vs. WT; °°° *P* < 0.001 vs. respective control without FA; §§§ *P* < 0.001 vs. respective control without MRS2500 and L-NAME. # *P* < 0.05, ### *P* < 0.001 vs. respective control with FA and without MRS2500 and L-NAME; *N* = 6 mice/group.

P2 receptors are necessary for the development of glial cells [39], and their activation in the CNS is associated to astrogliosis, whereas in the ENS they control connexin-43 hemichannels, causing glial-driven neuronal death [20]. Accordingly, we evaluated the immunofluorescence of connexin-43 in LMMPs of TLR4^−/−^ mice, which resulted comparable to that of WT mice and was not affected by FA treatment (Figure 6A,B). Finally, we evaluated the effect of FA treatment on the inhibitory response of ileal segments incubated with the pan-NOS inhibitor L-NAME and MRS2500. As shown in Figure 6C, in both genotypes pre-treatment with both inhibitors completely blocked EFS-evoked NANC relaxations. Conversely, after FA treatment, the relaxant response was only partially abolished, suggesting that the disruption of EGCs activity may reveal the presence of other underlying inhibitory neurotransmissions sustaining small intestine relaxation.

## 4. Discussion

FA and its metabolite FC are metabolic poisons commonly used to study glial cell functions since they cause the block of the catalytic activity of aconitase, an enzyme of the Krebs cycle and are preferentially taken up by glial cells [22,26]. Nasser et al. (2006) have shown that treatment with FA or FC could not change the glial phenotype and the number of total neurons in WT mice [23]. In this study, FA treatment in TLR4^−/−^ mice reduced S100β and GFAP immunofluorescence density, to levels comparable to WT. However, a higher total number of HuC/D^+^ neurons associated with the absence of changes in SOX10 immunoreactivity was observed in the myenteric plexus of FA-treated ileal segments of TLR4^−/−^ animals, suggesting the onset of adaptive neuroplastic changes following glia disruption [16,40].

SOX10 is one of the earliest neural crest cell biomarkers of ENS progenitors and its deletion causes the failure of enteric ganglia development, highlighting its role in ENS homeostasis [40]. During prenatal development, the expression of the transcription factor SOX10 in the ENS reveals the distribution of enteric neural precursor cells, which will generate neurons since SOX10^+^ cells have neurogenic activity during embryogenesis. In adult mice this neurogenic activity is lost and SOX10 is expressed by enteric glia [41]. However, Laranjeira et al. (2011) [40] demonstrated that under treatment with 4-hydroxytamoxifen mature EGCs can generate multilineage ENS progenitors and activate neurogenic programs. Neurogenesis is generally undetectable in the ENS of adult animals, but in response to injury, mature glial cells may generate enteric neurons [40]. In adult animals, a substantial phenotypic plasticity occurs among EGCs subtypes, as demonstrated by Boesmans et al. (2015) [16]. All subtypes of EGC originate from a common progenitor, which will generate distinct glial subtypes depending on its final destination and physiological context. The potential ability of glial cells to gain different properties is not restricted to embryonic or early postnatal stages, but is maintained throughout adult life, pointing to a previously unacknowledged dynamic phenotypic plasticity of the ENS, capable of adjusting its molecular characteristics in response to diverse challenges associated with nutrition, microbiota, mechanical factors, or disease [16]. EGCs communicate through Ca^2+^ signals, decoding and integrating information transmitted by neurons, immune cells, or other cells in the gut milieu [5]. Moreover, EGCs express neurotransmitter receptors, suggesting that, like astrocytes, they are active contributors in neuronal communication, and they can influence synaptic transmission [3].

**Figure 7:**
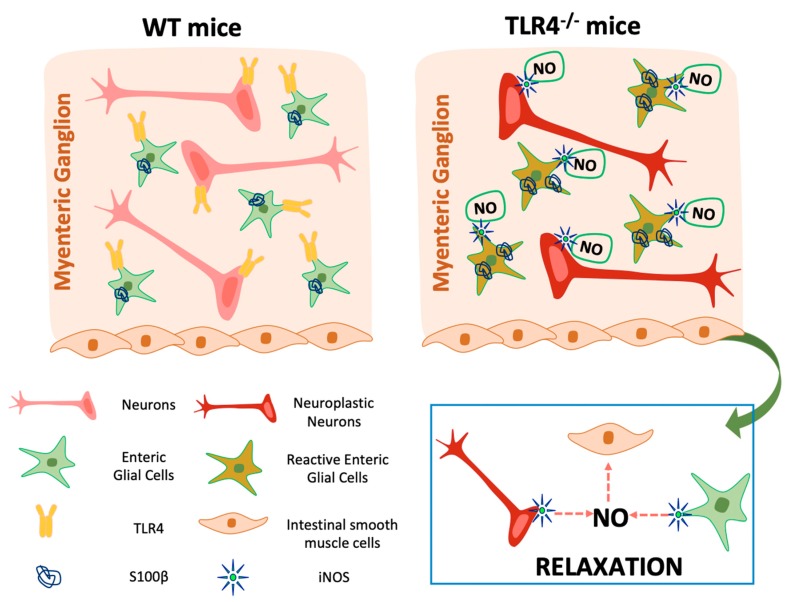
Graphical diagram depicting the influence of enteric glia on small intestine motility of TLR4^−/−^ mice.

In the ENS, the major inhibitory NANC neurotransmitter is endogenous NO that can be generated by the three different enzymes, nNOS, endothelial NOS (eNOS), and inducible NOS (iNOS). More than 90% of the total NOS in the small intestine is nNOS, expressed by nitrergic neurons, mostly distributed in the myenteric plexus, which act as inhibitory interneurons or inhibitory motoneurons [42]. However, iNOS isoform is also constitutively present and accounts for less than 10% of the total enteric NOS activity whereas eNOS isoform is barely detectable [42,43]. In case of inflammation, the induction of iNOS produces a large amount of NO in epithelial and immune cells as well as in neurons and EGCs of the ENS, determining altered epithelial function and water and ion transport dysregulation with consequent intestinal dysmotility [24,33,34,42,44,45,46]. Indeed, we have recently shown that, in NANC conditions, isolated ileal segments from TLR4^−/−^ mice display increased EFS-mediated relaxations associated with a proportional increase of nNOS^+^ neurons and an increase of both iNOS activity and immunoreactivity, whereas in WT mice the inhibitory tone was mainly dependent on nNOS-mediated NO production [29]. To better understand the role of EGCs in the neuromuscular function, we evaluated the effect of FA in the inhibitory response following 10 Hz EFS-stimulation. After FA treatment, in WT mice we found a slight but not significant reduction of inhibitory response to indicate that EGCs do not participate in the ileal relaxation during homeostasis, confirming the data found by Aubé et al. (2006) [47] in jejunal segments from a transgenic model of glial ablation. In TLR4^−/−^ mice, the disruption of EGC metabolism restored an inhibitory response comparable to WT mice, supporting a role for enteric glia in intestinal physiology. Moreover, iNOS activity in TLR4^−/−^ mice was abolished following in vitro FA treatment, confirming that TLR4 signaling is involved in controlling EGCs activity and iNOS expression. Accordingly, MacEachern et al. (2015) [24] showed that FA could prevent NO release specifically from iNOS, suggesting that FA directly targets enteric glial metabolism. Our current data suggest that, in the absence of TLR4 signaling, EGCs are in an activated state, leading to: (i) reactive gliosis (higher S100B content), (ii) neuroplastic changes (reduced number of HuC/D^+^ neurons), and (iii) release of iNOS-derived NO, principally from myenteric glia (Figure 7).

In both the large and small intestine, inhibitory motor neurons mediate smooth muscle relaxations by releasing NO and other neurotransmitters, such as ATP [38,48,49]. Among the purinergic receptors, P2Y1Rs is the one of the most important metabotropic receptor subtypes involved in inhibitory neurotransmission in the gastrointestinal tract [38,48,49]. We have recently shown that, in TLR4^−/−^ mice, P2Y1R immunoreactivity is increased in both neurons and glial cells of the small intestine myenteric plexus and these changes were associated with a higher inhibitory response of ileal segments [29]. interestingly, stimulation of P2Y1Rs in the CNS enhances reactive astrogliosis [50]. Therefore, we evaluated the activity of P2Y1R following FA-mediated inhibition of enteric glia activity. In both TLR4^−/−^ and WT mice, the combination of FA and of the highly potent and selective P2Y1 antagonist [51], MRS2500, determined a reduction of the inhibitory response comparable to the response obtained in presence of MRS2500 alone. P2Y1R immunoreactivity did not change in both the genotypes after FA treatment. These data indicate that the relaxation mediated by P2Y1R is mostly due to the activation of neural and not glial P2Y1R.

McClain et al. (2014) [52] showed that the activation of P2Y1R generates Ca^2+^ responses, leading to the opening of connexin-43 hemichannels. Connexin-43 hemichannels are necessary for the propagation of Ca^2+^ responses among glial cells, highlighting the role of EGCs in modulating gut smooth muscle contractility through connexin-43 activity [20,52]. In the CNS, astroglial connexin-43 hemichannels are involved in the release of neuroinflammatory mediators, contributing to the development of neuropathic pain and neurodegeneration [20]. In isolated preparations of mouse ENS, Brown et al. (2016) [20] demonstrated that the opening of connexin-43 hemichannels in glial cells, via direct activation of P2Y1R and concomitant presence NO, induces ATP release affecting neuronal survival leading to loss of neuronal density. Thus, we evaluated the inhibitory response of small intestinal preparations in presence of MRS2500 and L-NAME, a pan NOS inhibitor. In the absence of FA, blockade of P2Y1R and NOS in both genotypes, completely abolished the relaxation recorded at 10 Hz-EFS, highlighting the role of purinergic and nitrergic neurotransmissions in sustaining the principal inhibitory pathways in the gut [48]. However, in both genotypes, the inhibitory response was only partially abolished after FA treatment, suggesting that the disruption of EGCs activity may cause the involvement of other inhibitory pathways, including other purinergic receptors [49]. Indeed, following exposure to danger cues, different amounts of extracellular nucleotides (e.g., ATP, ADP, and UTP) and adenosine, generated from ATP hydrolysis, are rapidly released into the extracellular milieu and may influence enteric neuronal function via P2 and P1 receptors, respectively [53]. The fine tuning of synaptic activity by enteric glia may thus be an essential element of enteric neuronal signaling and motility. In the mouse ileum, beside P2Y1 receptors, the neurotransmitter ATP can induce muscular relaxation by activation of P2Y4 receptors. Indeed, recent studies have proposed that EGCs express P2Y4 receptors, which is involved in the purinergic neuron-to-glia signaling in the ENS [53,54,55]. Anomalies of purinergic neuron-to-glia signaling could be due to high ATP concentrations as evidenced by our previous demonstration of higher density of P2X7 receptors in enteric neurons [29] resulting in abnormal release of neuro/gliotransmitters or cytokines. In this respect, purines (e.g., ATP, adenosine) released by glia or neurons could influence ENS integrity via P2 and P1 receptors. However, this area of neurogastrointestinal research is still in its infancy [32,49].

In conclusion, the present findings strongly suggest that studies on EGCs are essential for elucidating their functional significance in the pathological conditions of the gastrointestinal tract, including inflammation and neurodegeneration [53,54,55].

## Figures and Tables

**Table 1:** Primary and secondary antisera and their respective dilutions used for immunohistochemistry on ileal whole-mount preparations.

Antibody	Host Species	Dilution	Catalog Number	Source
Primary Antisera (Clone)				
HuC/D (16A11)	Mouse biotin-conjugated	1:100	A-21272	Thermo Fisher Scientific (Monza, Italy)
nNOS (polyclonal)	Rabbit	1:100	61-700	Thermo Fisher Scientific
GFAP (polyclonal)	Chicken	1:100	ab4674	Abcam (Cambridge, UK)
S100β (EP1576Y)	Rabbit	1:50	ab52642	Abcam
iNOS (polyclonal)	Rabbit	1:100	sc-8310	Santa Cruz Biotechnology (Heidelberg, Germany)
P2Y1 (polyclonal)	Rabbit	1:50	APR-009	Alomone Labs (Jerusalem, Israel)
connexin-43 (polyclonal)	Rabbit	1:50	ACC-201	Alomone Labs
SOX10 (EPR4007)	Rabbit	1:50	ab155279	Abcam
Secondary Antisera				
Goat anti-rabbit IgG Alexa 488-conjugated	-	1:1000	A-11008	Thermo Fisher Scientific
Goat anti-chicken IgY Alexa 555-conjugated	-	1:1000	A-11039	Thermo Fisher Scientific
Streptavidin Alexa 555-conjugated	-	1:1000	S21381	Thermo Fisher Scientific

**Table 2:** Sequence of primers used for q-RT-PCR analysis.

Gene	Sequence 5′–3′
*HuC/D*	F-AAGAGTCCCCTGTCGCTCA R-TACACGAAGATGCACCAGCC
*S100β*	F-GACTCCAGCAGCAAAGGTGA R-ATCTTCGTCCAGCGTCTCCA
*iNOS*	F-CAGCTGGGCTGTACAAACCTT R-CATTGGAAGTGAAGCGTTTCG
*nNOS*	F-GTGGCCATCGTGTCCTACCATAC R-GTTTCGAGGCAGGTGGAAGCTA
*P2Y1R*	F-AGTGTGTGCCACCTGAGTGA R-ACCCTTGAGCTTGGAATGGAAT
*β-actin*	F-TGACAGGATGCAGAAGGAGA R-TAGAGCCACCAATCCACACA
